# Pulmonary Hypertension in Intensive Care Units: An Updated Review

**Published:** 2019-03

**Authors:** Armin Nowroozpoor, Majid Malekmohammad, Seyyed Reza Seyyedi, Seyed Mohammadreza Hashemian

**Affiliations:** 1 Clinical Tuberculosis and Epidemiology Research Center, NRITLD, Shahid Beheshti University of Medical Sciences, Tehran, Iran,; 2 Tracheal Diseases Research Center, National Research Institute of Tuberculosis and Lung Disease (NRITLD), Shahid Beheshti University of Medical Sciences, Tehran, Iran,; 3 Lung Transplantation Research Center, Department of Cardiology, National Research Institute of Tuberculosis and Lung Diseases (NRITLD), Shahid Beheshti University of Medical Sciences, Tehran, Iran

**Keywords:** Pulmonary hypertension, Pulmonary arterial hypertension, Intensive care, Critical care

## Abstract

Pulmonary hypertension (PH) is a condition associated with high morbidity and mortality. Patients with PH who require critical care usually have severe right ventricular (RV) dysfunction. Although different groups of PH have different etiologies, pulmonary vascular dysfunction is common in these groups. PH can lead to increased pulmonary artery pressure, which can ultimately cause RV failure. Clinicians should be familiar with the presentations of this disease and diagnostic tools. The contributing factors, if present (e.g., sepsis), and coexisting conditions (e.g., arrhythmias) should be identified and addressed accordingly. The preload should be optimized by fluid administration, diuretics, and dialysis, if necessary. On the other hand, the RV afterload should be reduced to improve the RV function with pulmonary vasodilators, such as prostacyclins, inhaled nitric oxide, and phosphodiesterase type 5 inhibitors, especially in group 1 PH. Inotropes are also used to improve RV contractility, and if inadequate, use of ventricular assist devices and extracorporeal life support should be considered in suitable candidates. Moreover, vasopressors should be used to maintain systemic blood pressure, albeit cautiously, as they increase the RV afterload. Measures should be also taken to ensure adequate oxygenation. However, mechanical ventilation is avoided in RV failure. In this study, we reviewed the pathophysiology, manifestations, diagnosis, monitoring, and management strategies of PH, especially in intensive care units.

## INTRODUCTION

Pulmonary hypertension (PH) is a group of disorders, characterized by increased pulmonary artery pressure (PAP) exceeding the physiological level. The mean PAP (mPAP) ≥25 mmHg, measured by right heart catheterization (RHC) at rest, has been used for the diagnosis of PH (1). PH is classified into five main groups according to similarities in clinical presentations, pathological findings, hemodynamic features, and management strategies. Updated classifications by the Fifth World Symposium on Pulmonary Hypertension are outlined in Table 1 (2).

**Table 1.** Causes of Pulmonary Hypertension in the Intensive Care Unit

**Cardiothoracic surgery**
Heart/Lung transplantation
Mitral valve surgery
Coronary artery bypass graft
Ventricular assist device placement
Pneumonectomy

**Deteriorating existing pulmonary hypertension**
Impaired medication use
Sepsis
Pneumonia
Acute on chronic pulmonary hypertension
Arrhythmias (esp. atrial fibrillation)

**Acute on chronic pulmonary hypertension**

**Pulmonary embolism**
Massive embolism
New emboli in CTEPH

**Parenchymal lung disease**
Acute respiratory distress syndrome

**Sepsis**

Patients with PH are admitted to the intensive care unit (ICU) for a variety of reasons (Table 2). ICU admission due to undiagnosed group 1 PH or pulmonary arterial hypertension (PAH) has decreased substantially as a result of improved knowledge about the disease (3). The majority of these patients are treated with PAH-specific drugs before ICU admission (4, 5). There are seven approved pharmacological agents targeting three major molecular pathways for the treatment of PAH (6), with newer drugs under development. Despite advances in management strategies and emergence of specific pharmacotherapies, PAH remains a challenging disease with high mortality.

**Table 2.** Classification of pulmonary hypertension (updated from Simonneau et al.(2))

**1. Pulmonary arterial hypertension**
1.1 Idiopathic PAH
1.2 Heritable PAH
1.2.1 BMPR2
1.2.2 ALK-1, ENG, SMAD9, CAV1, KCNK3
1.2.3 Unknown
1.3 Drug and toxin induced
1.4 Associated with:
1.4.1 Connective tissue disease
1.4.2 HIV infection
1.4.3 Portal hypertension
1.4.4 Congenital heart diseases
1.4.5 Schistosomiasis
1′ Pulmonary veno-occlusive disease and/or pulmonary capillary hemangiomatosis
1″. Persistent pulmonary hypertension of the newborn (PPHN)
**2. Pulmonary hypertension due to left heart disease**
2.1 Left ventricular systolic dysfunction
2.2 Left ventricular diastolic dysfunction
2.3 Valvular disease
2.4 Congenital/acquired left heart inflow/outflow tract obstruction and congenital cardiomyopathies

**3. Pulmonary hypertension due to lung diseases and/or hypoxia**
3.1 Chronic obstructive pulmonary disease
3.2 Interstitial lung disease
3.3 Other pulmonary diseases with mixed restrictive and obstructive pattern
3.4 Sleep-disordered breathing
3.5 Alveolar hypoventilation disorders
3.6 Chronic exposure to high altitude
3.7 Developmental lung diseases
**4. Chronic thromboembolic pulmonary hypertension (CTEPH)**

**5. Pulmonary hypertension with unclear multifactorial mechanisms**
5.1 Hematologic disorders: chronic hemolytic anemia, myeloproliferative disorders, splenectomy
5.2 Systemic disorders: sarcoidosis, pulmonary histiocytosis, lymphangioleiomyomatosis
5.3 Metabolic disorders: glycogen storage disease, Gaucher disease, thyroid disorders
5.4 Others: tumoral obstruction, fibrosing mediastinitis, chronic renal failure, segmental PH

BMPR = bone morphogenic protein receptor type II; CAV1 = caveolin-1; ENG = endoglin; HIV = human immunodeficiency virus; PAH = pulmonary arterial hypertension.

Survival of different PH groups (Table 1) and subgroups varies significantly (7, 8). Therefore, special attention must be paid to different mechanisms involved in the disease process. A retrospective cohort study by Saydain et al. on PH patients admitted to ICUs showed that severe PH, defined by right atrial pressure >20 mmHg, mPAP >55 mmHg, or cardiac index (CI) <2 L/min/m^2^, was a significant independent predictor of mortality. However, factors including an underlying disease, sepsis, or presence of comorbidities alone were not found to be predictors of mortality (5).

RV failure/dysfunction plays a vital role in the survival of patients with PH. However, there is no universal definition for this condition (7, 9). Several indices of RV failure seem to be well correlated with patient mortality (Table 2). Generally, RV failure is a major outcome of advanced PH, associated with high rates of mortality and morbidity. RV dysfunction/failure may occur in any of the five groups of PH, but it is mostly reported in patients with PAH (group 1) and chronic thromboembolic pulmonary hypertension (CTEPH; group 4) (4, 6, 7).

Despite significant advances in the knowledge of PH, there are still controversies about PH with RV dysfunction, especially in terms of management. Intensive care management of PH mainly relies on expert opinion, and there is a scarcity of high-quality randomized clinical trials (RCTs). In this review, we first briefly described the pathophysiology of RV dysfunction in PH patients and then focused on the diagnostic approaches and management strategies based on the available literature. It should be noted that PH in pediatric patients was not examined.

### Pathophysiology of right ventricle dysfunction in pulmonary hypertension

Although different groups of PH vary in terms of pathogenesis, there are still some similarities. Pathogenesis of PH includes three main pathways: 1) increased production of endothelins; 2) decreased production of prostacyclins; and 3) decreased production of nitric oxide (NO). Endothelin-1, as a potent vasoconstrictor, increases in patients with PH. This has been well established both in animal and human models of PH (10–14). However, it is not clear if it plays a causal role in the disease or is a consequence of it (6, 15–17). Endothelin-1 also increases smooth muscle cell proliferation and has mitogenic effects (18, 19).

Prostacyclin is a potent vasodilator, which acts by increasing the level of cyclic adenosine monophosphate. A decrease in the production of prostacyclin has been reported in patients with PAH. Prostacyclin also reduces vascular remodeling, inflammation, platelet aggregation, and thrombosis. Thromboxane, a potent vasoconstrictor, increases due to PAH, and an imbalance in prostacyclin/thromboxane production is a proposed mechanism in the development of PAH (19–24). NO, which is produced by endothelial cells, acts by increasing cyclic guanosine monophosphate (cGMP) to cause vasodilation. Decreased production of NP due to endothelial dysfunction has been shown to play a role in PH (25–27). Treatment of PH, especially PAH as the most studied variant of PH, focuses on different targets of these three major pathways. Currently, there are nine drugs in four classes, approved for the treatment of PAH. These classes include prostanoids, soluble guanylate cyclase stimulators, endothelin receptor antagonists (ERAs), and phosphodiesterase type 5 (PDE5) inhibitors (6, 28).

Normal pulmonary circulation is a so-called “high-flow, low-pressure” system. Its maintenance requires the reduction of pulmonary vascular resistance (PVR) in response to increased blood flow. Disruption in the normal vasodilatory reserve of pulmonary vessels and pulmonary vascular dysfunction are known to interrupt this system. The relationship between pressure, flow, and resistance is understandable based on the following equation:

PVR=[(mPAP-PCWP)/CO]×80

where PVR represents the pulmonary vascular resistance, mPAP is the mean pulmonary artery pressure, PCWP is the pulmonary capillary wedge pressure, and CO is the cardiac output. Due to the great potential of pulmonary vessels in vasodilation (ability of PVR to decrease significantly), an increased flow (measured as CO in the equation) does not lead to a considerable increase in PAP in physiological conditions. However, when this potential is compromised, the increased flow causes PAP to rise.

As described earlier, there are multiple etiologies for PH, but RV dysfunction and ultimately RV failure are common in severely ill patients. It is well established that RV function is an important predictor of survival in patients with PH (7, 9). As a result, RV failure should be emphasized in the treatment of patients with PH in ICUs. Most studies on ventricular failure are restricted to the LV. However, some findings can be also true for the right ventricle (RV). The RV, compared to the LV, has a thinner wall and a more complex geometry. From the front view, it is somewhat triangular (compared to the conical LV), and from the cross-sectional view, it is crescent-shaped. It has a convex free wall and a concave interventricular septum (29–31). Based on the Laplace’s law (see below), wall stress in a spherical or cylindrical chamber is directly proportionate to the internal radius of the chamber and intraluminal pressure and inversely proportionate to the wall thickness: $\sigma \propto \frac{P.r}{h}$

where *σ* represents the wall tension, *P* denotes the RV pressure, r represents the internal radius of RV, and *h* is the wall thickness of RV. According to this equation, wall tension increases by increasing the intraluminal pressure. To prevent an excessive increase in wall tension, either the internal radius of RV should decrease or wall thickness of RV should increase. Wall tension is a good correlate of RV afterload. RV, due to its thin wall and low volume/surface area, shows greater compliance than the muscular thick-walled LV (LV).

**Figure 1.**
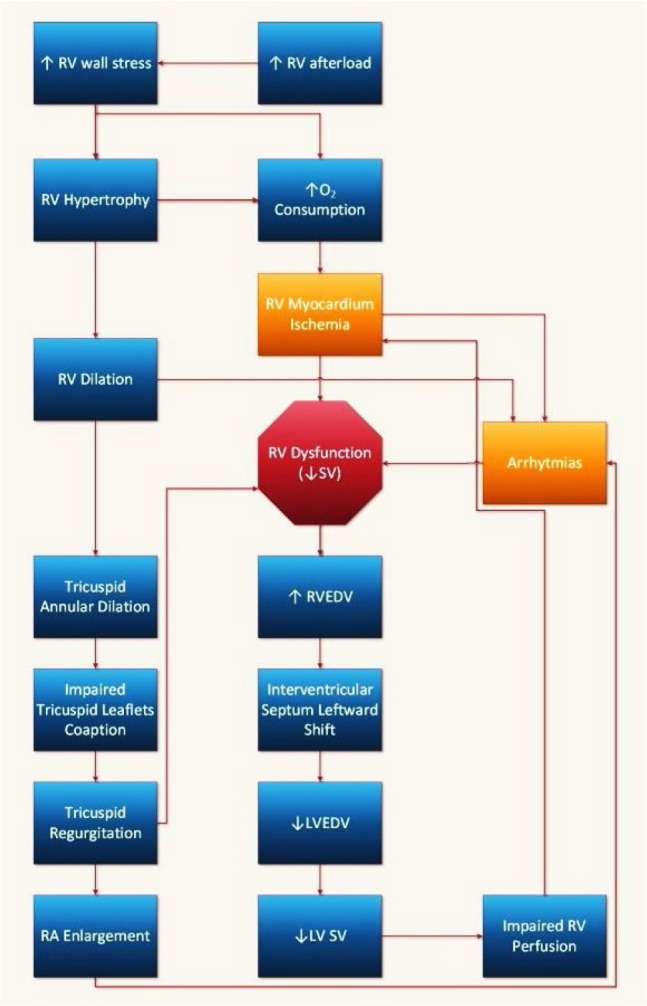
Pathophysiologic cycle of right ventricle dysfunction in pulmonary hypertension. LV= left ventricle; LVEDV= left ventricle end-diastolic volume; RA= right atrium; RV= right ventricle; RVEDV= right ventricle end-diastolic volume; SV= stroke volume

Due to the increased RV afterload in PH, the RV wall hypertrophies as a physiological response to reduced wall tension. This process is considered a compensatory mechanism to oppose the increased wall tension (i.e., to decrease the RV wall tension). These adaptive mechanisms, however, cannot compensate for the sustained RV pressure overload, and the contractile force of RV does not suffice, leading to RV dilation and RV dysfunction. Increased RV mass also leads to increased myocardial oxygen demand (29, 32–34) (Figure 1).

LV is affected by RV due to ventricular interdependence. In other words, the anatomical and functional characteristics of one ventricle can affect the anatomical and functional characteristics of another (35–38). RV dysfunction causes an increase in the RV enddiastolic volume (RVEDV), which causes the interventricular septum to shift towards the LV. It also increases the pericardial constraint of LV during diastole. These changes decrease the LV end-diastolic volume (37, 38). Abnormal (paradoxical) septal motion may also impede LV ejection, reducing the LV flow during systole (37). This results in the diminished RV blood supply, which together with the increased oxygen demand of the hypertrophied RV (explained above), further deteriorates the oxygen supply/demand balance and leads to a more severe RV dysfunction (32, 37) (Figure 1).

### Evaluation of patients with pulmonary hypertension in Intensive Care Units

#### Clinical manifestations

Most symptoms and signs of PH are non-specific, which causes a delay in diagnosis and initiation of treatment, especially in idiopathic PAH (39, 40). The symptoms may include dyspnea (especially on exertion), chest pain, coughs, palpitations, fatigue, orthopnea, paroxysmal nocturnal dyspnea, syncope, near-syncope, and peripheral edema. Abdominal pain, distension, and early satiety may also occur due to RV failure, causing hepatic congestion (41, 42). Moreover, hoarseness may occur with compression of the left recurrent laryngeal nerve due to a dilated pulmonary artery (Ortner’s syndrome) (43). Hemoptysis rarely occurs, but if present, it is usually associated with PAH (44, 45) or concomitant pulmonary embolism, pulmonary infarction, or severe mitral stenosis (41).

Physical examination generally reveals signs of decompensated RV failure, including jugular vein distention, hepatomegaly, ascites, and peripheral pitting edema. Other signs may include a left parasternal lift, accentuated P2 sound, pansystolic murmurs due to tricuspid regurgitation, diastolic murmurs due to pulmonary insufficiency, S3 and S4 gallops, cooling of extremities, and diminished pulse pressure. Some signs may suggest a specific cause of PH; for example, the presence of venous stasis ulcers may suggest sickle cell disease (6, 46, 47).

### Diagnostic tools and monitoring of pulmonary hypertension in intensive care units

Several tools may be used to improve the evaluation of patients with PH, including electrocardiography (ECG), echocardiography, chest X-ray, computed tomography (CT), RHC, and more recently, cardiac magnetic resonance (CMR) imaging and positron emitting tomography (PET). Moreover, laboratory markers may be helpful in identifying the specific causes of PH, monitoring of treatment, and patient prognosis.

### ECG analysis

ECG remains an essential part of PH assessment. However, it is neither a specific nor a sensitive screening/diagnostic tool for PH. The RV strain has been shown to have more sensitivity for this purpose (48). A synthesized right-sided ECG seems to be more accurate in detecting PH; however, use of this type of ECG is less common (49). Positive findings are more likely to be found in severe PH and may include right axis deviation, RV hypertrophy, RV strain, P pulmonale, right bundle branch block, increased QTc interval, and QRS prolongation (6). Supraventricular tachyarrhythmias (SVTs) are the main dysrhythmias in PH. Atrial flutter, atrial fibrillation, and AV-nodal reentrant tachycardia are the most common types. These conditions have a poor prognosis and further deteriorate the already impaired CO, leading to a rapid clinical decline (6, 50–52).

### Chest X-ray

Chest X-ray is not a very suitable tool for the diagnosis of PH in the ICU setting, but it may reveal signs correlated with the disease severity or underlying factors. Signs, such as pulmonary arterial enlargement, RV hypertrophy, and an obscure aortopulmonary window, may not be observable on a portable image. Other signs which may help establish an underlying cause include pleural effusion and septal Kerley B lines (suggesting left heart disease), flat diaphragms and hyperinflated lungs (suggesting chronic obstructive pulmonary disease), and spine and chest wall abnormalities (suggesting restrictive pulmonary disease) (46, 53).

### Echocardiography

Transthoracic echocardiography (TTE) with the use of Doppler ultrasound is the most available and widely used tool for diagnosing and monitoring patients with PH. Although it may not be an accurate tool, especially in critically ill patients, it provides valuable information for clinicians. It can effectively identify congenital heart diseases, such as intracardiac shunts or left heart disease as the underlying cause of PH (54). Moreover, it can estimate the right atrial pressure (RAP), mPAP, and level of RV dysfunction and help determine the underlying cause of PH non-invasively (55).

To estimate mPAP, TTE uses functional tricuspid regurgitation (TR), which occurs as a result of disturbance in the geometrical structure of RV in PH; therefore, this measurement is prone to over- or underestimation. Furthermore, an increase or decrease in mPAP is not necessarily correlated with the clinical status or prognosis of patients (6). Signs indicating a dysfunctional RV include RV and RA hypertrophies, dyskinesia of the right free wall of RV (McConnell’s sign), straightening or leftward bowing of the interventricular septum (D-shaped LV), paradoxical movement of the septum in the systole, and a dilated inferior vena cava (53, 56, 57).

Evaluation of the RV size and function may be challenging due to its crescent shape and thin walls. Several parameters have been used to assess the RV function, which seem to be well correlated with the disease prognosis, including estimated RAP, RV longitudinal strain, tricuspid annular plane systolic excursion, right ventricular Tei index (myocardial performance index), RV fractional area change, and RV strain based on speckle tracking (6, 58–65). Other parameters that can be used at bedside to evaluate the RV function may include the RV/LV area ratio, LV eccentricity index, peak velocity of systolic excursion, and RV systolic pressure (66).

Three-dimensional (3D) echocardiography is emerging as a useful tool for the assessment of RV. It can accurately estimate the RV size and ejection fraction (EF) and improve the assessment of RV function non-invasively (67). The findings of 3D echocardiography are well correlated with cardiac MRI, and therefore, it is reasonable to use it as a non-invasive and accurate tool for the evaluation of RV function in combination with 2D echocardiography (68–70).

### Computed tomography scan

Non-contrast CT scan is a readily available tool, which provides useful information about mediastinal structures, such as heart chambers, larger vessels, lung parenchyma, and bony structures. It can depict signs on the chest X-ray more accurately and help with the diagnosis of PH and its underlying causes. High-resolution CT may be especially helpful in evaluating the lung parenchyma and detecting interstitial lung disease, pulmonary veno-occlusive disease, and pulmonary capillary hemangiomatosis. This modality is now recommended for all patients with PH (6, 71).

CT pulmonary angiography (CTPA) is a valuable tool in detecting arteriovenous malformations, pulmonary embolism, and CTEPH, with a specificity as high as 99% (72). Several indices have been proposed for diagnosing PH and assessing the disease severity, such as main pulmonary artery diameter and pulmonary artery-to-aorta ratio (73, 74). ECG-gated CTPA is associated with fewer motion-related artifacts and allows for the assessment of coronary arteries simultaneously (75, 76). The newer “dual-energy CT” has also shown comparable accuracy in detecting CTEPH and may help decide about the efficacy of CTEPH (75, 77, 78). However, conventional pulmonary angiography remains the gold standard for diagnosing patients who are suitable candidates for surgical interventions (i.e., pulmonary endarterectomy or balloon pulmonary angioplasty) (6).

### Ventilation/perfusion scintigraphy

Ventilation/perfusion scintigraphy (V/Q scan) is the most sensitive screening tool of choice for detecting pulmonary embolism and CTEPH. A normal or a very-low-probability V/Q scan essentially rules out CTEPH. CTPA is more specific, but less sensitive than V/Q scans (6, 72). However, some studies have shown that CTPA is as sensitive and accurate as V/Q scan in detecting CTEPH (79). Definite diagnosis of CTEPH should be made through CTPA, conventional contrast angiography, or MR angiography (6).

### MRI studies

Cardiovascular magnetic resonance (CMR) imaging is an accurate and reliable modality and the gold standard for the non-invasive assessment of RV size, geometry, and function (80); however, its use in the ICU setting is limited. It has been shown that estimation of mPAP, based on the ventricular mass index, is well correlated with RHC measurements (81). Nevertheless, it is not as accurate as RHC, and it is only used for monitoring patients and treatment response. By the measurement of the RV stroke volume (SV) and RV volume, CMR can detect RV dysfunction before it is clinically evident (80). Increased RV volume, decreased RV-SV and RV end-diastolic index (RVEDVI), and reduced LV volume seem to be associated with treatment failure and mortality (82, 83).

Magnetic resonance angiography (MRA) is used to differentiate idiopathic pulmonary arterial hypertension (IPAH) and CTEPH (84). MRA may be also helpful in diagnosing congenital heart disease if Doppler echocardiography is inconclusive. In addition, it can be used as an alternative to CT angiography in case of iodine contrast contraindication (85). Moreover, hybrid PET-MRI is a novel technique, which allows for both structural and functional assessments of heart chambers and may shed more light on the pathophysiology of RV dysfunction. Furthermore, it may play multiple roles in the assessment of RV in patients with PH in the future (86).

### Right heart catheterization

RHC is still considered the gold standard for the definite diagnosis of PH and analysis of vasoreactivity in patients with IPAH, heritable pulmonary arterial hypertension (HPAH), or drug-induced PAH, which may benefit from calcium channel blockers (6). However, application of RHC in the critical care setting has been debated. Many observational studies have found no improvement in the clinical outcomes of patients undergoing RHC for monitoring. RHC has been even associated with increased mortality and resource utilization (87, 88). Some RCTs comparing the management of critically ill patients with or without RHC found no significant difference regarding its benefits or harms (89–92). Moreover, a recent Cochrane meta-analysis comparing the same outcomes concluded the same results (93).

Different assessment methods (e.g., Fick’s method and thermodilution method) of CO with RHC may provide different results. Serious complications include arrhythmias, pneumothorax, hematoma at the insertion site, systemic hypotension, infection, and air or clot embolism. However, if performed by skilled experts, the morbidity and mortality associated with this procedure are relatively low (1.1% for the overall rate of serious adverse outcomes and <0.1% for fatal complications) (94). Extra caution must be exercised when performing RHC in patients with IPAH, HPAH, and PAH (due to the use of anorexigens) and patients with arrhythmias, as they may be receiving oral anticoagulants.

Based on these findings, use of RHC as a routine monitoring technique in critically ill patients has declined over the years due to its high cost, infrequent but serious complications, need for high expertise, and emergence of newer technologies. However, many experts still advocate for the use of RHC in patients with PH and severe RV dysfunction in the critical care setting (4, 53). Therefore, it is reasonable to decide about RHC on a case-by-case basis with regard to the patient’s clinical condition, physician’s expertise, and technical availability.

### Other measures

Routine hematology, biochemistry, and thyroid function tests are required for all patients. Serial creatinine measurement should be performed, especially if the patient’s condition is deteriorating. Renal function is associated with increased mortality (95). Serological tests are required to diagnose the underlying cause of PH if previously undetermined. Arterial blood gas should be obtained to assess the partial pressure of oxygen (PaO _2 _) and partial pressure of carbon dioxide (PaCO _2 _) to guide management. Lung diffusion capacity for carbon monoxide is decreased in pulmonary veno-occlusive disease, scleroderma, and interstitial lung disease (6). Monitoring of coagulation status is essential, especially in those receiving anticoagulation therapy.

Many biochemical markers have been studied for the detection, prognosis, and treatment response of patients with PH. Brain natriuretic peptide (BNP), a cardiac hormone, and N-terminal pro-brain natriuretic peptide (NT-proBNP), its inactive alternative, are the most important studied markers. Both BNP and NT-proBNP are elevated as a result of increased ventricular wall tension. They are recognized as markers of dysfunction in both ventricles; therefore, they are not specific to RV (96, 97). BNP/NT-proBNP is correlated with mPAP (98). Moreover, the level of NT-proBNP during treatment is correlated with RVEDVI, RV mass index, RV-EF (99), and PVR (100). The prognostic values of both BNP and NT-proBNP are well established (101–104). Changes in the level of these markers represent the treatment response and can guide management although they are not suitable tools for diagnosing RV dysfunction.

### Management

Management of PH patients with RV dysfunction and decompensated RV failure has not been studied exclusively in critical care settings, and there is no standard guideline. In this section, we mainly focus on managing RV dysfunction caused by PH in critical care settings. General management strategies for PH are provided in detail by the 2015 European Society of Cardiology (ESC)/European Respiratory Society (ERS) guidelines (6). According to the Frank-Starling law, SV of a ventricle is dependent on three factors: 1) preload, 2) afterload, and 3) inotropy (contractility). With a constant afterload and inotropy, SV is determined by preload.

Dilation leads to increased sarcomere length in physiological limits, resulting in increased contractile force. In a chronically volume-overloaded RV, SV is relatively preserved with hypertrophy of RV. However, with further volume overload or acute volume/pressure overload of the RV (e.g., acute pulmonary embolism), RV fails to maintain a normal SV. This is primarily due to the increased length of sarcomeres beyond their physiological limit, which results in decreased contractility (36). In this section, management strategies will be discussed in three main categories: optimization of 1) RV preload, 2) RV afterload, and 3) RV contractility (36, 105). Instead of a step-wise approach, all three categories must be considered simultaneously as they are intricately related.

### Right ventricle preload

Assessment of the patient’s volume status is a very important and challenging issue, especially in critical care. The ultimate goal of physicians is to provide adequate and effective intravascular volume. This is necessary for effective perfusion of both systemic and pulmonary circulations. Fluid therapy is a controversial topic in the management of RV failure. It is difficult to define the optimal goal of RV preload in patients with RV failure, and it is somewhat unknown whether increasing the RV preload has beneficial effects in a failing RV.

RAP, RVEDV, and RVEDP are usually considered as surrogates for the RV preload (30). In a 1999 study on patients with acute massive pulmonary embolism and normal systemic pressure, fluid loading was shown to increase RAP, RVEDVI, stroke volume index, and CI; however, it did not affect EF or PVR (106). An excess increase in preload causes more ventricular dilation, resulting in increased wall stress, ischemia, and leftward interventricular septal shift (see pathophysiology above) and deterioration of RV and LV function (Figure 1).

Many experts advocate for modest fluid loading in patients suspected of having a low preload (36, 107, 108). It should be performed with initial assessment and continuous monitoring, such as using a central venous line; however, it should not continue if clinical improvement does not occur (36, 109). With a central venous pressure (CVP) ≥12–15 mmHg, fluid loading should be withheld, and measures should be taken to decrease the afterload and increase the inotropy (108).

Two measures that can improve the assessment of fluid responsiveness include passive leg raising (PLR) and respiratory variations in thoracic pressure. PLR is essentially useful in patients who are neither on vasoconstrictors nor mechanically ventilated. A positive PLR suggests that the patient will respond to fluid loading (110–112). Changes in pulse pressure in mechanically ventilated patients can predict fluid responsiveness. However, this technique cannot be used in patients with dysrhythmias or tachycardia (110).

Most cases of RV failure involve volume overload and require diuretics or hemodialysis to remove the excess fluid (4, 109). The amount of fluid needed to be removed may be substantial for significant improvement of RV function (105). An optimal RV filling pressure of 8–12 mmHg has been suggested by some authors (105, 109). Achieving a normal superior vena cava O _2_ saturation of 70–80% can be also used to guide the amount of fluid removal, as it is a marker of CO (105). Regarding diuretics, a loop diuretic, such as furosemide, is usually used to remove the excess fluid. A continuous infusion is preferred, as it is titratable to achieve optimal filling pressures. If response to high-dose loop diuretics is not favorable, a thiazide diuretic can be added (107). If the diuretic response is still inadequate, the patient may undergo hemodialysis or ultrafiltration. Echocardiography may also guide the management of fluids, either fluid loading or removal, by comparing the indices of RV function before and after therapy (66) (see echocardiography above).

### Right ventricle afterload

Increased RV afterload is an important component of PH and RV failure. Pulmonary vasodilators and PAH-specific pharmacotherapy play major roles in managing these patients since RV is especially vulnerable to increased afterload. Prostacyclins, inhaled NO, ERAs, and PDE5Is are the available options, although there is a scarcity of high-quality RCTs on the management of these patients in critical care settings. PAH-specific drugs should be used with caution in PH groups other than PAH, as they may not be effective and may have potential complications (107, 113, 114). PAH-specific drugs may be beneficial in inoperable CTEPH and perioperative PH (6, 115, 116). It should be noted that many previously diagnosed PAH patients may be already receiving a combination of PAH-specific drugs, which is an increasingly common practice.

### Prostacyclins

Prostacyclins are the mainstay treatment for PAH and severe PH with RV dysfunction. Their effects may be the greatest in patients without previous prostacyclin treatment and those with a more severe disease. Intravascular infusion of epoprostenol or treprostinil is usually used as the initial treatment (117). Dosing starts at 1–2 ng/kg/min and then increases by 1–2 ng/kg/min every 15–30 minutes for epoprostenol and by 5 ng/kg/min for treprostinil. Dosing increases gradually until a favorable hemodynamic response or maximum patient tolerability is achieved. Epoprostenol, due to its shorter half-life (3–6 minutes) than treprostinil (2–4 hours), may be theoretically a better option in critical care settings. Treprostinil can be also administered as a subcutaneous infusion with the same dosage and similar effects, although pain at the site of infusion may be a major setback (118–122). Patients receiving subcutaneous treprostinil may be transitioned to intravenous epoprostenol (preferred) or intravenous treprostinil, with dose adjustments in the ICU (123, 124).

Attention must be paid to potential systemic vasodilation and hypotension with systemic prostacyclins. Inhaled prostacyclins are less potent in inducing systemic hypertension. Since they are only delivered to ventilated alveoli, they can theoretically reduce the V/Q mismatch (105). Inhaled epoprostenol, which has been used in a few studies, increases the CI and mixed venous oxygen saturation and reduces mPAP and CVP, without decreasing the systemic arterial pressure (115, 116, 125). Also, some limited studies have shown the favorable outcomes of inhaled epoprostenol in acute respiratory distress syndrome (ARDS) and perioperative PH and RV failure (115, 126–130).

Inhaled treprostinil, starting at 18 mcg four times a day, improved the quality of life and NT-proBNP in a 12-week double-blind RCT on PAH patients already on sildenafil or bosentan (131). In a previous study, the combined use of inhaled treprostinil and sildenafil in patients with PAH and CTEPH caused a significant reduction in PVR and mPAP and increased CO (132). Other studies have also shown the favorable hemodynamic effects and minor side effects of inhaled treprostinil in PH patients (133–138). It is known that inhaled iloprost decreases PVR, mPAP, mean arterial pressure (MAP), and PaO _2_ in patients with CTEPH (139). Long-term inhaled iloprost at 2.5–5 mcg (6–9 times per day) has been also used to treat severe PH (140). It has been also administered in patients undergoing cardiothoracic surgery, mitral valve surgery, and cardiopulmonary bypass (115, 141, 142).

Selexipag is a novel selective prostacyclin receptor agonist, approved for the treatment of PAH (143). When used in combination with ERAs and/or PDE5Is, it can reduce PVR and mPAP and increase CO and CI; however, it has not been shown to reduce mortality (144, 145). Use of selexipag in critically ill patients has not been studied, and it should not be initiated in a critical care setting if the patient is not already receiving it.

### Nitric oxide (NO)

Inhaled NO (iNO) has become a routine part of the management of patients with ARDS and many patients with PH and RV failure. Similar to inhaled prostacyclins, iNO exerts its effects on well-ventilated areas of the lung and improves the V/Q mismatch (146). It is known that iNO undergoes rapid metabolism by hemoglobin in the pulmonary system, and its very short half-life requires continuous administration. Risk of methemoglobinemia, albeit low, increases by increasing the dose and prolonging the use (147). It has been shown that iNO reduces mPAP and PVR, increases the RV-EF, and improves oxygenation in patients with ARDS and PH; however, these outcomes are mostly transient. In this regard, a recent meta-analysis showed that iNO does not result in reduced mortality or decreased length of hospital/ICU stay and may even increase the risk of renal failure (148).

Despite the fact that all prostacyclins can improve the hemodynamic parameters, only epoprostenol seems to reduce long-term mortality in patients with idiopathic PAH (121, 149, 150). In a previous study, inhaled epoprostenol and iNO resulted in similar reductions in mPAP among patients with PH undergoing thoracic surgery. Both groups had a similar duration of mechanical ventilation, hospital stay, ICU stay, and in-hospital mortality, although the cost of inhaled epoprostenol was significantly lower than iNO (151). Both iNO and prostacyclins should not be discontinued abruptly, as they may cause rebound PH and RV failure (152, 153).

### Phosphodiesterase 5 inhibitors (DE5Is)

PDE5Is increase the cGMP level, cause vasodilation, and reduce smooth muscle cell proliferation, mPAP, and PVR (154, 155). Sildenafil and tadalafil are FDA-approved drugs for the treatment of PAH, which cause vasodilation; their peak effects are seen 60 and 75–90 minutes after administration, respectively (156). They both exhibit selectivity for pulmonary vasculature although systemic hypotension may occur (156). Only sildenafil has been shown to increase arterial oxygenation (156).

Sildenafil with iNO exerts synergistic hemodynamic effects and prevents rebound PH seen in iNO withdrawal (157–162). Sildenafil has been used preoperatively in patients with severe PH undergoing surgery. Sildenafil has been also used in combination with prostacyclins and ERAs, causing greater improvements in the hemodynamics (132, 163–169). Oral sildenafil has a rapid onset (15–30 minutes) and a short duration of action (4–6 hours). It is suitable for use in ICUs (107), although its intravenous form is also available (170–172).

### Right ventricle contractility

In a failing RV, improvement of contractility should be considered. Drugs used to decrease the RV afterload, such as intravenous prostacyclins, can also increase the RV contractility (173). Studies comparing different inotropic and vasopressor agents, especially in PH patients, are scarce. Milrinone, as an inodilator, is a phosphodiesterase-3 (PDE-3) inhibitor, which increases cAMP, leading to vasodilation and increased contractility (174–176). In addition, it can improve oxygenation, decrease PVR, and increase RV-SV (176, 177). Milrinone can cause systemic hypotension and increase the need for vasopressors. However, removal of the bolus dose before infusion may prevent these outcomes (178–180).

Intravenous administration of milrinone in combination with iNO has shown greater effectiveness than their administration alone (181, 182). Inhaled milrinone may be used to avoid adverse effects, such as systemic hypotension (183–185). In a study of PH following cardiac surgery, inhaled milrinone in combination with inhaled epoprostenol could decrease mPAP and PVR and increase RV-SV (186). Therefore, intravenous milrinone should be used with caution and preferentially as the last resort. Milrinone can also help prevent rebound hypertension due to iNO withdrawal by preserving the cAMP levels (187).

Although dopamine increases CI, it can cause tachycardia and increase the PVR/SVR ratio (36). Its effects may come at the cost of increased arrhythmic events and mortality in patients with cardiogenic shock (36, 188, 189). Dobutamine improves hemodynamics in patients with PH undergoing liver transplantation. It also improves RV infarction-induced PH, as well as PH in heart failure patients with preserved EF (190–192). In a previous study, administration of low-dose dopamine and/or dobutamine (starting at 3 mcg/kg/min) at the onset of epoprostenol treatment was a safe option in PAH patients and did not increase mPAP (193). At doses higher than 5 mcg/kg/min, dobutamine may cause systemic hypotension and require the use of a vasopressor, such as norepinephrine (53). Dobutamine (10 mcg/kg/min) in combination with iNO can increase CI and PaO _2_ and decrease PVR and SVR, without changing the mPAP or MAP (194). Therefore, many clinicians prefer using dobutamine over dopamine (6, 107).

Levosimendan is a calcium sensitizer, which increases myocardial contractility unlike ß-adrenergic agonists and PDE-3 inhibitors, without increasing the intracellular calcium. It also causes pulmonary and coronary vasodilation (195, 196). It has the advantage of not increasing myocardial O _2_ consumption and even decreasing it (195). In animal models of PH, levosimendan has been shown to increase the RV contractility, SV, CI, and RV-PA coupling, while decreasing PVR (197–199). Levosimendan may be associated with an increased need for norepinephrine infusion (200, 201). Studies on the effects of levosimendan on mortality have yielded inconclusive results (202). Short-term use of levosimendan in patients with PH and RV failure, especially in case of biventricular failure, may be considered (36). Epinephrine increased SV, CI, and MAP, but did not increase PVR in animal models of acute PH (203–205). In a small pilot open-label clinical trial on pediatric patients with PH, epinephrine increased the aortic pressure and mPAP and exerted inconsistent effects on the PVR/SVR ratio in different patients (206).

### Vasopressors

In addition to dopamine and epinephrine, other vasopressors include norepinephrine, phenylephrine, and arginine vasopressin. In case of systemic hypotension, use of a suitable vasopressor is required. Furthermore, maintaining the aortic root pressure is vital for adequate coronary perfusion in both RV and LV. The ideal vasopressor is one that increases SVR to maintain MAP, without increasing PVR, which results in an unfavorable increase in mPAP.

Norepinephrine exerts its effect through α-1 and β-1 stimulation (207). It causes systemic and pulmonary vasoconstriction through α-1 stimulation, thereby increasing SVR and PVR (208). However, as shown in animal studies and one human case report, low doses of norepinephrine are usually used in clinical settings (<0.5 mcg/kg/min), and an increase in PVR is less likely (209, 210). Due to its inotropic effects, norepinephrine increases the RV contractility, RV-PA coupling, and CO (209, 211–213). In chronic PH and RVF, norepinephrine decreases the PVR/SVR ratio in patients with normal CI (214).

Phenylephrine at low doses only activates α-1 receptors and does not have β sympathetic activity (207). In patients with chronic PH, it increases SVRI, PVRI, MAP, and mPAP, but it does not change mPAP/MAP or PVRI/SVRI ratio. At higher doses, phenylephrine decreases CI, compared to norepinephrine. Although phenylephrine may improve the coronary blood flow, it may have deleterious effects on the RV function and CO (204, 212, 215); therefore, its use is usually limited.

Arginine vasopressin (AVP) is a potent vasopressor, exerting its effects through V1 receptors on vascular smooth muscle cells (207). AVP causes systemic vasoconstriction, while at low doses, it causes selective pulmonary vasodilation, resulting in decreased or preserved PVR and PVR/SVR ratio (216–221). However, there is conflicting information regarding its pulmonary vasodilatory role and effects on RV function (219, 222, 223); also, at higher doses, it seems to produce pulmonary vasoconstriction (224). Additionally, AVP may have age-dependent effects, as it does not cause pulmonary vasodilation in neonate rats (225). AVP may be useful in decreasing the required dose for concurrent vasopressor use in patients with septic shock (219, 226–228), although no favorable effects on mortality have been reported (226). There are some case-reports on the clinical use of AVP in acute (229) and chronic PH (230–232). However, more studies are needed for a better understanding of its effect in this population.

### Oxygenation and mechanical ventilation

Adequate oxygenation is of utmost importance in ICU patients with PH. Alveolar and arterial hypoxia, acidosis, and hypercapnia can all cause pulmonary vasoconstriction (36), which in turn may exacerbate these conditions; therefore, measures should be taken to end this cycle. Oxygen saturation should be kept at >90–92% (105, 233, 234). It should be noted that supplemental oxygen decreases the heart rate, mPAP, and CO (235). Both hyperinflation and atelectasis decrease the alveolar blood flow and increase PVR. The effects on PVR are minimal at functional residual capacity (36, 109). Increased intrathoracic pressure, which occurs with positive pressure ventilation, increases the RV afterload and decreases the RV-EF (236). Intubation should be avoided in cases of RV failure (6) for as long as possible, as it is associated with increased in-hospital mortality. In a large cohort study of patients with PAH, the in-hospital mortality was 39.1% in patients undergoing invasive mechanical ventilation versus 12.1% in those undergoing non-invasive mechanical ventilation (P<0.001). Overall, high-flow oxygen, administered through the nasal cannula, may be a safer option (237).

Many authors have discouraged permissive hypercapnia in patients with PH (4, 53, 107) as increased CO _2_ levels may increase PVR and mPAP and deteriorate RV function due to pulmonary vasoconstriction (238, 239). This vasoconstriction may be reversed using iNO (240). In contrast, some animal studies have found a protective role for CO _2_ in PH induced by chronic hypoxia (241, 242). It may be reasonable to consider PaCO _2_ of 48–50 mmHg as the upper limit, as it is considered for patients with ARDS (243). Moderate hypercapnia (PaCO _2_ = 60–70 mmHg) may be tolerated in selected cases (244).

Other lung-protective ventilation strategies for ARDS, which may be reasonable in patients with PH and RV failure, include lower tidal volume (<6 ml/kg of ideal body weight), optimization of PEEP (≤10 cm H _2_ O if clinically feasible) (245), lower plateau pressure (<30 cm H _2_ O) (246, 247), and consequently lower driving pressure (248, 249). However, these decisions should be made on a case-by-case basis, as an individual’s response to different ventilation mechanisms varies (250). Newly emerging extracorporeal CO _2_ removal devices may allow for the use of even lower tidal volumes (<4 ml/kg of ideal body weight) and PEEPs in the future (ultra-protective ventilation) (251).

Extracorporeal membrane oxygenation (ECMO) may be used either as a bridge to transplantation or as a bridge to recovery in decompensated PH and RV failure until other therapeutic measures are applied to manage the condition. Veno-arterial ECMO must be used rather than veno-venous ECMO as it can unload RV in addition to providing oxygenation (252). Application of ECMO, pre-, intra-, and post-lung transplant may have favorable effects on cardiac function and mortality and allow for earlier weaning from mechanical ventilation postoperatively (253, 254).

Left-, right- and bi-ventricular assist devices (LVADs, RVADs, and BiVADs) may be used in patients with severe ventricular dysfunction. A failing RV may help with the insertion of RVAD to improve the RV function. Improvement of RV function comes at the expense of increased PVR, PAP, and damage to the pulmonary microcirculation, leading to pulmonary hemorrhage; this is more prominent with pulsatile flow, compared to constant flow systems (255, 256). Increased duration (>7 days) and flow of RVAD (>4 L/min) (257) are associated with increased mortality.

Many patients require RVAD insertion due to RV failure after LVAD placement or BiVAD placement due to biventricular failure (258–262). There are reports of isolated RVAD placement for RVF in patients with RV infarction, orthotopic heart transplant (263–268), and RVF with PH (269–274). Percutaneous RVADs are effective and do not require sternotomy (269, 275). A pumpless lung assist device acts as an artificial lung and may be used as a bridge to lung transplant in patients with PAH. It connects the pulmonary artery trunk to the left atrium, with blood passing through a membrane oxygenator. It can unload the RV by bypassing the high resistance pulmonary vasculature. Although it is effective in removing CO _2_ , it does not provide significant or adequate oxygenation. Additionally, the blood flow exclusively relies on the pressure gradient from the pulmonary artery to the left atrium (276–278). In this regard, Vasanthan et al. reported a patient on a pumpless lung assist device for 82 days before undergoing heart and bilateral lung transplantation (279).

### Arrhythmias

SVTs, including atrial flutter and atrial fibrillation, are the most prevalent arrhythmias in PAH, while ventricular arrhythmias are rare. SVTs are associated with impaired RV function, clinical deterioration, and increased mortality in case of atrial fibrillation, while restoring the sinus rhythm improves the RV function. Rhythm control is more important in PAH, while rate control is more important in non-PAH patients with LVF (52). Electric cardioversion and ablation therapy are both safe and effective in rhythm control. Sinus rhythm should be maintained using antiarrhythmic drugs, without any major negative inotropic activity (6). Bradycardia in PAH is alarming as it is highly associated with cardiopulmonary arrest (52). Oral anticoagulation should be initiated in all patients with SVTs (6).

### Other measures

Graded balloon dilation atrial septostomy may be performed only in specialized centers as palliative care or a bridge to transplantation when medical therapies have failed (280, 281). It creates a right-to-left shunt, bypassing the lungs, unloading the RV, and increasing the LV preload and CO. Although oxygenation decreases, tissue oxygen delivery improves. This procedure should not be performed in patients with very severe PH with a mean baseline RAP >20 mmHg and O _2_ saturation <85% at rest in the room air (6). Stent fenestration of the septum may be performed to prevent the closure of the foramen (282). Intra-aortic balloon counterpulsation is used in case of severe heart failure to improve the LV function by decreasing the LV preload, afterload, and oxygen demand (283) and to increase the coronary blood flow if coronary autoregulation is impaired (283, 284). Use of this method has been reported in animal models of RV failure (285–287) following cardiopulmonary bypass (288, 289) and heart transplant (290, 291).

## CONCLUSION

Management of PH in the ICU setting is challenging for clinicians. In patient care, the type of PH, its pathophysiology, underlying exacerbating factor, and individual patient characteristics must be taken into account since there is no clear understanding or consensus on many aspects of management. In addition, the exacerbating factor (e.g., infection and anemia) should be identified and treated promptly. Principal measures, such as optimizing the RV afterload, preload, and contractility, should be also addressed. PAH-specific therapies continue to evolve over time, with newer medications and findings changing the practice. Assessment of the volume status is critical, mandating fluid therapy and/or diuretics along with other measures. Moreover, RV contractility must be improved using appropriate drugs if the patient’s findings suggest RV failure. Pulmonary vasodilators are a promising option for decreasing the RV afterload. However, maintaining the systemic blood pressure with vasopressors is challenging as they eventually increase PAP. Palliative or bridging strategies, such as mechanical support, should be also considered in certain candidates. Future studies should address the uncertainties surrounding this rare, but fatal disease.
